# Genetic Polymorphism of CYP2C19 in Pakistani Population

**DOI:** 10.22037/ijpr.2019.1100644

**Published:** 2019

**Authors:** Sana Riaz, Sadia Muhammad Din, Muhammad Usman Tareen, Fizza Tariq, Yusra Latif, Saima Siddiqi, Aneesa Sultan, Atika Mansoor

**Affiliations:** a *Institute of Biomedical and Genetic Engineering, 24 Mauve Area, G-9/1, Islamabad,* *Pakistan.*; b *Department of Biochemistry, Faculty of Biological Sciences, Quaid-i-Azam University, Islamabad,* *Pakistan.*

**Keywords:** Genotyping, Adverse drug reactions, CYP2C19, Pakistanis, Single nucleotide polymorphisms, Ethnic groups

## Abstract

CYP2C19 polymorphism is associated with pretreatment drug response prediction, metabolism, and disposition. Pakistan consists of a population comprising of various ethnic groups residing in different regions of the country each claiming diverse ethnic origins. The identification of CYP450 genotypic composition of these populations is therefore necessary to avoid adverse drug reactions in these individuals. The main objective of the study was to investigate the prevalence of CYP2C19*2 and CYP2C19*17 alleles in these ethnic groups. The study was conducted on one thousand and twenty-eight (n = 1028) healthy volunteers from nine ethnic groups of Pakistan namely Brusho (n = 28), Hazara (n = 102), Kalash (n = 64), Pathan (n = 170), Punjabi (n = 218), Saraiki (n = 59), Brahui (n = 118), Parsi (n = 90), and Sindhi (n = 179). DNA was extracted from leukocytes and analyzed by allele specific amplification polymerase chain reaction (ASA-PCR). Multi allelic polymorphism of CYP2C19 led to four distinct phenotypes identified as extensive metabolizer (EM), poor metabolizer (PM), intermediate metabolizer (IM), and ultra-rapid metabolizer (UM). Over all, the percentage of predicted poor metabolizer allele was 29.0% compared to UM allele (23.70%). Among the studied groups, Saraiki and Brahui showed highest percentage of PM allele (40%, 36%) whereas Parsi and Hazara had highest percentage of UM allele (37% and 30% respectively). In conclusion, the high allele frequency of PM (CYP2C19*2 and *17) in Pakistani population led to the recommendation of a pre-treatment test to monitor drug response and dosage (personalized medicine) to avoid post-treatment adverse drug reactions.

## Introduction

Adverse drug reactions (ADRs) and failure to therapeutics represent a major health problem and constitute a big challenge for drug development. The enzymatic transformation of therapeutically important compounds into active, inactive or more soluble forms is catabolized by cytochrome P450 system (1). CYP1, 2, and 3 are involved in the metabolism of more than 75% clinically used drugs; CYP2C19 being a part of CYP2 family is mainly involved in the metabolism of Proton Pump Inhibitors (PPI) and antiepileptics. Omeprazole the most widely sold PPI is included in the WHO Essential list of medicines. Apart from PPI some antiplatelet drugs such as clopidogrel, antiepileptic (mephenytoin), and antimalarial (proguanil) are also metabolized by CYP2C19.

CYP2C19 has more than twenty variant alleles but most important *2, *3, *4, *5, *6, *7, and *8 are associated with reduced enzyme function (2). CYP2C19*1 represents the wild type allele and its homozygotes are known as extensive metabolizers. CYP2C19*2 is the most important variant formed due to the splice site defect in exon 5 (single base change G > A) of the gene and is marked for loss of function allele. It accounts for more than 90% of CYP2C19 poor metabolizers and individual with its homozygous defective alleles are termed as PM (3). Apart from loss of function alleles, a promoter variant CYP2C19*17 of CYP2C19 leads to increased activity of the gene and is classified as ultra-rapid metabolizer. Individuals with CYP2C19*17 both heterozygous and homozygous form are rapid metabolizers (4). 

About 10-20% of patients treated with PPIs are nonresponders to the treatment. Frequency of CYP2C19 poor and rapid metabolizers varies worldwide; Asians (12-23%) and East Asian (14-39%) are reported to have highest occurrence of poor metabolizers (5, 6). Also, Africans (24%) and Europeans (22%) have higher frequency of rapid metabolizers (http://asia.ensembl.org). Diverse pharmacogenetic properties of individuals play an important role in failure to the treatment; therefore, it is imperative to know the individual’s genotype before starting any treatment. 

Pakistan consists of a population comprising of various ethnic groups residing in different regions of the country each claiming diverse ethnic origin. Therefore, it is very important to know the CYP450 genotypic composition of these populations in order to avoid adverse drug reactions in these individuals. The present study was planned to explore various Pakistani ethnic groups for prevalence of two most common allelic variants CYP2C19*2 and CYP2C19*17 involved in the poor or rapid metabolism of some of the important drugs.

## Experimental

*Study population*

The study population included one thousand and twenty-eight (n = 1028) unrelated healthy Pakistani subjects. These samples were collected from nine ethnic groups including Brusho (n = 28), Hazara (n = 102), Kalash (n = 64), Pathan (n = 170), Punjabi (n = 218), Saraiki (n = 59), Brahui (n = 118), Parsi (n = 90), and Sindhi (n = 179). A written informed consent was obtained from all subjects before participation in the study. Blood samples (5 mL in tubes containing EDTA) were collected from the subjects and DNA was extracted from peripheral blood leukocytes by phenol chloroform method (7). The study was approved by the Ethical Committee of Institute of Biomedical and Genetic Engineering (IBGE), Islamabad and the Institutional Review Board (IRB) of Quaid-i-Azam University, Islamabad. 

*Genotyping*

CYP2C19 alleles CYP2C19*2 (c.G681A; rs4244285) and CYP2C19*17 (c.C806T; rs12248560) were genotyped by ASA-PCR and the primer sequences are given in supplementary data (Table S1A and S1B). The amplified products were detected on 2% agarose gels for genotyping. Each PCR was carried out in 20 µL volume using 1.5 mM MgCl_2_, 50 mM KCl, 200 µM dNTPs, 10 mM Tris-HCl, 0.5 µM of each forward and reverse primer, 1 units of Taq DNA polymerase (Fermentas), and 100 ng of DNA sample. PCR amplification of CYP2C19*2 region was carried out by initial denaturation at 95 °C for 5 min, followed by 30 cycles of denaturation at 94 °C for 1 min, annealing at 62 °C for the amplification of “A” allele-specific DNA fragment and 60 °C for “G” allele-specific DNA fragment for 40 sec, extension at 72 °C for 1 min followed by final extension at 72 °C for 5 min. Thermal profile for the amplification of CYP2C19*17 was similar to CYP2C19*2 amplification except annealing at 58 °C for both alleles. The phenotypes were inferred from the genotypes. 

## Results

Our findings (Table 1) demonstrated the genotyping results for nine ethnic groups of Pakistan. The individuals were classified as extensive metabolizers (EM), intermediate metabolizers (IM), ultrarapid metabolizers (UM), and poor metabolizers (PM) depending on their genotype: EM had both functional alleles (*1/*1), IM had one functional and one loss of functional allele or one nonfunctional allele and one increased activity allele (*1/*2, *2/*17), UM carried both increased activity allele or one normal and one increased activity allele (*17/*17, *1/*17) and PM were those with both nonfunctional alleles (*2/*2) (8). 

**Figure 1:**
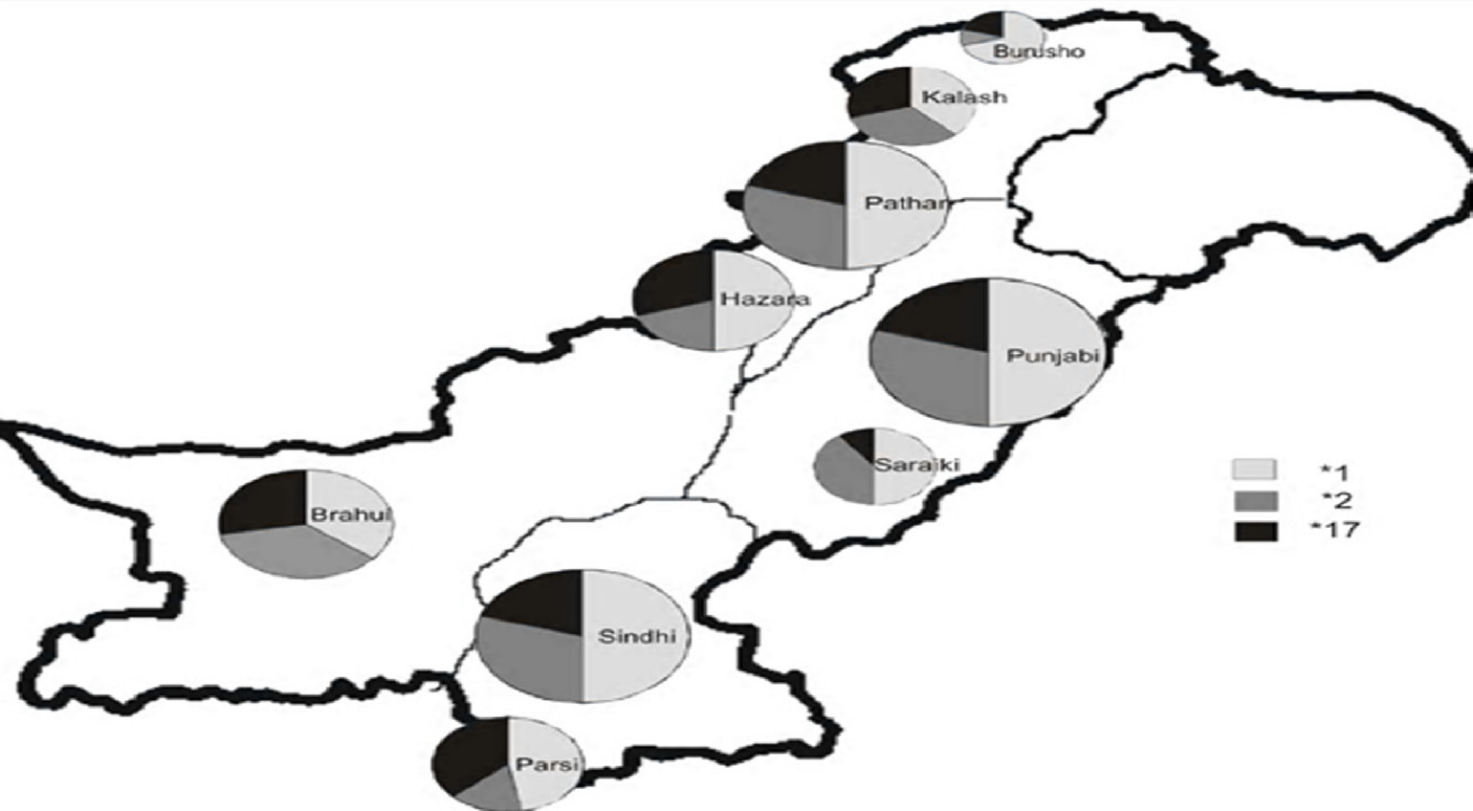
Prevalence of CYP2C19 alleles among nine Pakistani ethnic groups

**Figure 2:**
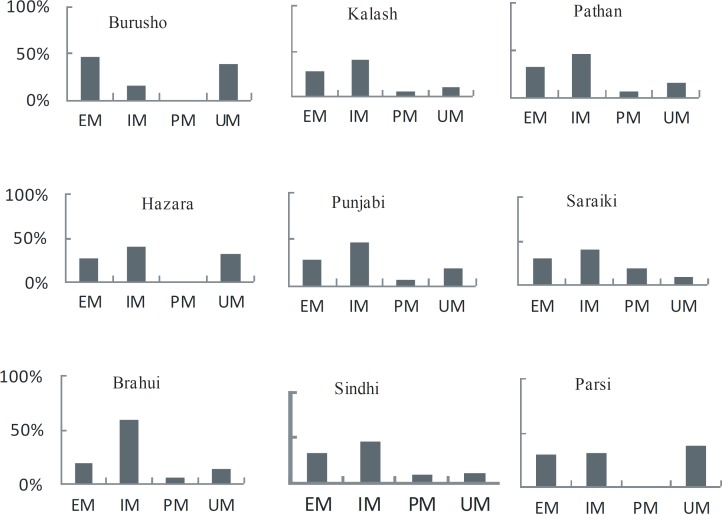
Predicted CYP2C19 phenotypes from genotypes in nine Pakistani ethnic groups

Among all the groups studied, CYP2C19*1 allele was predominant with allele frequency varying from 36% to 70% (Table 1 and Figure 1). However, the genotype frequency for extensive metabolizers (*1/*1) varied from 20% to 46% with an average of 30% for all ethnic groups. The overall frequency of PM allele for all groups was 29% with Burusho showing the lowest (10%) and the Saraikis showing the highest frequency (40%). The PM genotype (*2/*2) was highest in Saraikis (20%) whereas, in other ethnic groups it varied from 0 to 9%. Burusho and Parsi did not show any PM genotype. UM allele frequency averaged to 24% with highest in Parsi and lowest in Saraiki ethnic groups. At genotype level, the overall frequency of UM was 18% and it ranged from 10% to 39%. The frequency of IM was quite high about 46% for all populations with Brahui having the highest frequency (60%). At population level, IM genotype frequency was higher for most of the populations except Burusho and Parsi; Burusho had higher EM genotype and Parsi had UM as highest genotype (Figure 2). Our observation for the allele frequencies of *2 and *17 in Pakistani population was similar to that reported previously. Over all the percentage of predicted PM allele was present in 29.0% individuals compared to UM allele which was 23.70%.

## Discussion

Advancement in the field of Pharmacology has tremendous potential for management of various ailments. At the same time adverse drug reaction or drug-drug interaction can lead to various complications. CYP450 system plays an important role in the metabolism of pharmacologically active compounds; CYP2C19 accounts for bio-transformations of most of the PPI and antiepileptics.

Globally the pattern of transformations through CYP2C19 varies; Pakistan is situated on the southern coastal route from Africa to Australia (9). It has at least 18 different ethnic groups who speak >60 languages, mostly Indo-European languages but there are also some language isolates including Burushaski, Brahui, and Balti (9). Among the nine populations studied Burusho, Kalash, Hazara, and Pathan are found in Khyber Pukhtoonkhwa (KPK) province in the northern region of the country. Punjabi and Saraiki are in Punjab, Brahui in Baluchistan, and Parsi and Sindhi in Sindh province, in the south of Pakistan. Kalash, Burusho, and Pathan claim Greek origin whereas Hazara look like Mongols. Punjabi and Sindhi populations have admixture of other ethnic groups however Parsi have Iranian origin. The Brahuis have more complex origin, that is Indo-Iranian from Central Asia to East (9-11). The Saraikis depict historic pre-Aryan people of a Semite origin (12). Most of the studies have supported the genetic diversity among Pakistani populations and genetic clusters lie close to European populations (13, 14). 

**Table 1:** Predicted phenotypes for various Pakistani Ethnic Groups (n = 1028)

		**North Populations**					**South Populations**				
**GENOTYPE**	**Predicted**	**Burushu**	**Hazara**	**Kalash**	**Pathan**	**Punjabi**	**Saraiki**	**Buruhi**	**Parsi**	**Sindhi**	
	**Phenotype**	**(n = 28)**	**(n = 102)**	**(n = 64)**	**(n = 170)**	**(n = 218)**	**(n = 59)**	**(n = 118)**	**(n = 90)**	**(n = 179)**	**Total**
*1/*1	EM	13 (46%)	28 (27%)	18 (28%)	54 (32%)	60 (28.0%)	19 (30%)	24 (20%)	27 (30%)	63 (35.20%)	306 (29.80%)
*1/*17	UM	11 (39%)	30 (29%)	5 (8%)	22 (13%)	34 (16%)	5 (10%)	14 (12%)	26 (29%)	19 (10.60%)	166 (16.10%)
*2/*17	IM	1 (4%)	22 (22%)	29 (45%)	48 (28%)	53 (24%)	6 (10%)	49 (41%)	24 (26%)	42 (23.50%)	274 (26.70%)
*1/*2	IM	3 (11.0%)	19 (19%)	7 (11%)	31 (18.0%)	49 (22%)	19 (30%)	22 (19%)	5 (6%)	39 (21.80%)	194 (18.90%)
*2/*2	PM	0 (0%)	0 (0%)	3 (5%)	12 (7%)	16 (7%)	10 (20%)	7 (6%)	0 (0%)	16 (8.90%)	64 (6.20%)
*17/*17	UM	0 (0%)	3 (3%)	2 (3%)	3 (2%)	6 (3%)	0 (0%)	2 (2%)	8 (9%)	0 (0%)	24 (2.30%)
ALLELE											
*1	EM	40 (70%)	105 (50%)	48(37.5%)	161 (50%)	203 (50%)	62 (50%)	84 (35.6%)	85 (47%)	184 (50%)	972 (47.30%)
*2	PM	4 (10%)	41 (20%)	42(32.8%)	103 (30%)	134 (30%)	45 (40%)	85 (36%)	29 (16%)	113 (30%)	596 (29.00%)
*17	UM	12 (20%)	58 (30%)	38(29.7%)	76 (20%)	99 (20%)	11 (10%)	67 (28.4%)	66 (37%)	61 (20%)	488 (23.70%)

CYP2C19 polymorphism study was planned because of its importance in many cardiovascular, antiepileptic drugs and due to high prevalence of poor metabolizers in European and Asian populations. This type of study is important to avoid adverse drug reactions (ADRs) and extra financial burden on patients. CYP2C19 enzyme is responsible for inter-individuals and inter-ethnic differences within ethnic groups and between other ethnic groups in the world. The CYP2C19*2 allelic frequency in Pakistan is similar to other Asian countries namely Orientals, Thai, Korean, Japanese, and North Indians (15-19). Overall Asians represent high frequency of *2 allele compared to other countries (20). Allelic frequency of CYP2C19*17 in Pakistan is 23.7% which is similar to most of the Europeans including Danish, Norway, and Germany (21, 22). Among Asians the frequency is mostly lower except Saudi Arabia where it is 25.7% (23). From the world-wide distribution of CYP2C19*2 allele frequency it is obvious that it has relatively higher frequency compared to other variants showing this detrimental mutation is relatively older and occurred before the Black, Oriental and Caucasian racial groups split” (24, 25). Population frequency shown in this study will be helpful to recommend CYP2C19 (*2 and *17) typing as a pre-treatment test to monitor drug dosage (personalized medicine) to avoid ADRs as a personalized medicine approach. The study results might allow us in future to detect genetic variations in drug-metabolizing enzymes which are useful for clinicians to suggest right dosage and efficacy of drugs metabolized by this polymorphic enzyme to avoid adverse drugs reactions.

## Conclusion

CYP450 genotyping helps to identify those individuals who are at the risk of ADRs with routine doses of some commonly used drugs. Thus, clinicians can prescribe the right drug and acceptable dosage for the patients which in turn can prevent discomfort of taking unnecessary medicines and extra financial burden on the patients.
