# Supplementary material for: Genetic Polymorphism of CYP2C19 in Pakistani Population
Source: Iran J Pharm Res. 2019 Spring;18(2):1097–102. doi: 10.22037/ijpr.2019.1100644 (PMC6706708; doi:10.22037/ijpr.2019.1100644)
Supplement: Supplement [file ijpr-18-1097-s001.pdf]

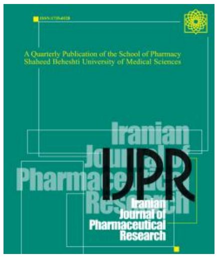

Supplementary Materials for

Genetic Polymorphism of CYP2C19 in Pakistani Population

Sana Riaz, Sadia Muhammad Din, Muhammad Usman Tareen, Fizza Tariq,
Yusra Latif, Saima Siddiqi, Aneesa Sultan and Atika Mansoor*

*To whom correspondence should be addressed. E-mail: atikamansoor@hotmail.com

Volume 18, Issue 2 (Spring 2019)

This PDF file includes:

Tables S1A and S1B

Table S1A. Primers for CYP2C19*2.

Primers	Sequence (5′-3′)	Length	Melting Temperature	Fragment Size (bp)	Type of PCR Product
2C19*2 F	CAGAGCTTGGCAATATTGTATC	22	57.1 °C	291	Control
2C19*2 R	ATACGCAAGCAGTCACATAAC	21	57.4 °C		
2C19*2 A	GTAATTTGTTATGGGTTCCT	20	52.3 °C	169	A allele fragment
2C19*2F	CAGAGCTTGGCAATATTGTATC	22	57.1 °C		
2C19*2G	ACTATCATTGATTATTTCCCG	21	55.6 °C	202	G allele fragment
2C19*2R	ATACGCAAGCAGTCACATAAC	21	57.4 °C		

Table S1B. Primers for CYP2C19*17.

Primers	Sequence (5′-3′)	Length	Melting Temperature	Fragment Size (bp)	Type of PCR Product
2C19*17F	AAGAAGCCTTAGTTTCTCAAG	21	55.5	507	Control
2C19*17R	AAACACCTTTACCATTTAACCC	22	56.6		
2C19*17T	TGTCTTCTGTTCTCAAAGTA	20	52.3	218	T allele fragment
2C19*17 R	AAACACCTTTACCATTTAACCC	22	56.6		
2C19*17 F	AAGAAGCCTTAGTTTCTCAAG	21	55.5	330	C allele fragment
2C19*17C	ATTATCTCTTACATCAGAGATG	22	54.7		
